# LIPUS as a potential strategy for periodontitis treatment: A review of the mechanisms

**DOI:** 10.3389/fbioe.2023.1018012

**Published:** 2023-02-22

**Authors:** Maierhaba Aimaijiang, Yiping Liu, Zhiying Zhang, Qiuyue Qin, Manxuan Liu, Palizi Abulikemu, Lijun Liu, Yanmin Zhou

**Affiliations:** Jilin Provincial Key Laboratory of Tooth Development and Bone Remodeling, Hospital of Stomatology, Jilin University, Changchun, China

**Keywords:** low-intensity pulsed ultrasound, periodontal inflammation, alveolar bone, mechanism, bone regeneration, mechanotransduction

## Abstract

Periodontitis is a chronic inflammatory condition triggered by oral bacteria. A sustained inflammatory state in periodontitis could eventually destroy the alveolar bone. The key objective of periodontal therapy is to terminate the inflammatory process and reconstruct the periodontal tissues. The traditional Guided tissue regeneration (GTR) procedure has unstable results due to multiple factors such as the inflammatory environment, the immune response caused by the implant, and the operator’s technique. Low-intensity pulsed ultrasound (LIPUS), as acoustic energy, transmits the mechanical signals to the target tissue to provide non-invasive physical stimulation. LIPUS has positive effects in promoting bone regeneration, soft-tissue regeneration, inflammation inhibition, and neuromodulation. LIPUS can maintain and regenerate alveolar bone during an inflammatory state by suppressing the expression of inflammatory factors. LIPUS also affects the cellular behavior of periodontal ligament cells (PDLCs), thereby protecting the regenerative potential of bone tissue in an inflammatory state. However, the underlying mechanisms of the LIPUS therapy are still yet to be summarized. The goal of this review is to outline the potential cellular and molecular mechanisms of periodontitis-related LIPUS therapy, as well as to explain how LIPUS manages to transmit mechanical stimulation into the signaling pathway to achieve inflammatory control and periodontal bone regeneration.

## 1 Introduction

Periodontitis is one of the most common chronic inflammatory non-communicable diseases among humans. The local inflammatory state caused by periodontitis leads to the progressive destruction of the periodontal supporting structures (gingiva, alveolar bone, and periodontal ligament), which may even lead to the loss of teeth (Kinane et al., 2017). Periodontitis is the inflammatory outcome of dysbiosis of the oral microbiota (dental plaque) and the interaction of such microorganisms with the host’s immune defense system (Pihlstrom et al., 2005; Pan et al., 2019a). According to recently developed clinical practice guideline by the European Federation of Periodontology, the treatments of periodontitis aim at controlling the supragingival and subgingival biofilm and calculus, local or systemic anti-inflammatory treatment, or further regeneration of periodontal loss or resection of lesions to minimize the complexity of periodontitis treatment (West et al., 2021; Herrera et al., 2022). As periodontitis develops, the balance between bone formation and bone resorption in the alveolar bone is disrupted by the state of chronic inflammation (Usui et al., 2021). Cell populations such as PDLCs, which contain stem cells with multiple differentiation capacities, experience prolonged ischemia, and hypoxia, depriving the periodontium of its regenerative microenvironment (Wang et al., 2020; Liu et al., 2021). GTR and bone grafting are currently the more commonly used methods to address periodontal bone loss. However, GTR remains unpredictable and clinical outcomes vary depending on the challenge of the host’s intraoral microbial biofilm, multiple risk factors (e.g., diabetes, smoking, dental plaque, and contact between the bone graft and non-vascular tooth surface), and the skill and experience of the practitioner (Vaquette et al., 2018; Liang et al., 2020). If there was a treatment strategy that targeted the pathogenesis of periodontitis, it could more effectively address the tissue loss due to inflammation.

The growth of new tissue and organs requires mechanical signals (Vining and Mooney, 2017; Steppe et al., 2020; Guo et al., 2021). Ultrasound stimulation appeared as a safe and non-invasive mechanical stimulation and has shown promise as a treatment for tissue regeneration (Daeschler et al., 2018; de Lucas et al., 2020; Liao et al., 2021). LIPUS is already widely used both in various clinical applications and fundamental research (Xin et al., 2016; Xie et al., 2019; Elvey et al., 2020). It has been acknowledged as a non-invasive physical stimulation for therapeutic purposes, delivering acoustic energy to the targeted tissue (de Lucas et al., 2021; Ichijo et al., 2021; Watanabe et al., 2021). Unlike high-intensity ultrasound, LIPUS has drawn attention for its minimal thermal effects (Xin et al., 2016). LIPUS is usually generated by a piezoelectric sensor, which converts electrical energy into mechanical force. The periodic acoustic waves that LIPUS generated can cause vibration and collision in the target tissue through the medium. In the process, cavitation, acoustic flow, and mechanical stimulation, as the main non-thermal effects of LIPUS, generate microbubbles and microjets to achieve the results (Lin et al., 2016). This non-thermal mechanism may also be explained by the influence of acoustic streaming during the action of LIPUS on the tissue. This acoustic flow may alter the local microenvironment of the cells, affecting the potassium content and calcium content of the cells after ultrasound exposure (Tanaka et al., 2015). Generally, the output frequencies of LIPUS range from 1 to 3 MHz. The ultrasound intensity of LIPUS therapy is within the range of 0.02–1 W/cm^2^ spatial average temporal average (SATA) and the treatment duration is 5–30 min per day (Kaur and El-Bialy, 2020; Al-Dboush et al., 2021).

LIPUS has also been used in clinical trials for fracture healing, and compelling clinical data demonstrate its effectiveness in bone fracture healing, especially in some high-risk non-union cases that are not suitable for surgery (Zura et al., 2015a; Rutten et al., 2016; Leighton et al., 2017; Bhan et al., 2021). Former studies have demonstrated that LIPUS is responsible for accelerating bone reparations through both upregulating osteogenic-specific genes and downregulating osteoclast differentiation (Costa et al., 2018; Meng et al., 2018). LIPUS also effectively provides the mechanical force beneficial to soft tissue regeneration (Lai et al., 2021), regulating inflammation and neuromodulation (Xu et al., 2021; Song et al., 2022). Studies have shown that LIPUS has several positive effects on dentofacial remodeling, especially in root and alveolar bone resorption after orthodontic treatment. Kasahara et al. (2017) found in a controlled experiment that LIPUS stimulation decreased the atrophic alterations of periodontal structures associated with the lack of functional occlusion after orthodontic treatment. Interestingly, LIPUS produces opposite impacts on osteoblasts and osteoclasts during orthodontic movements, thus significantly reducing root resorption without compromising tooth movement (Inubushi et al., 2013; Feres et al., 2016). Diabetes also harms the effectiveness of orthodontic treatment, including a reduction in bone regeneration on the tension side and a decrease in the number of PDLCs. However, LIPUS can ensure the efficacy of orthodontic treatment by increasing the bone remodeling on the tension side and the number of bone resorption traps on the compression side (Alshihah et al., 2020).

**FIGURE 1:**
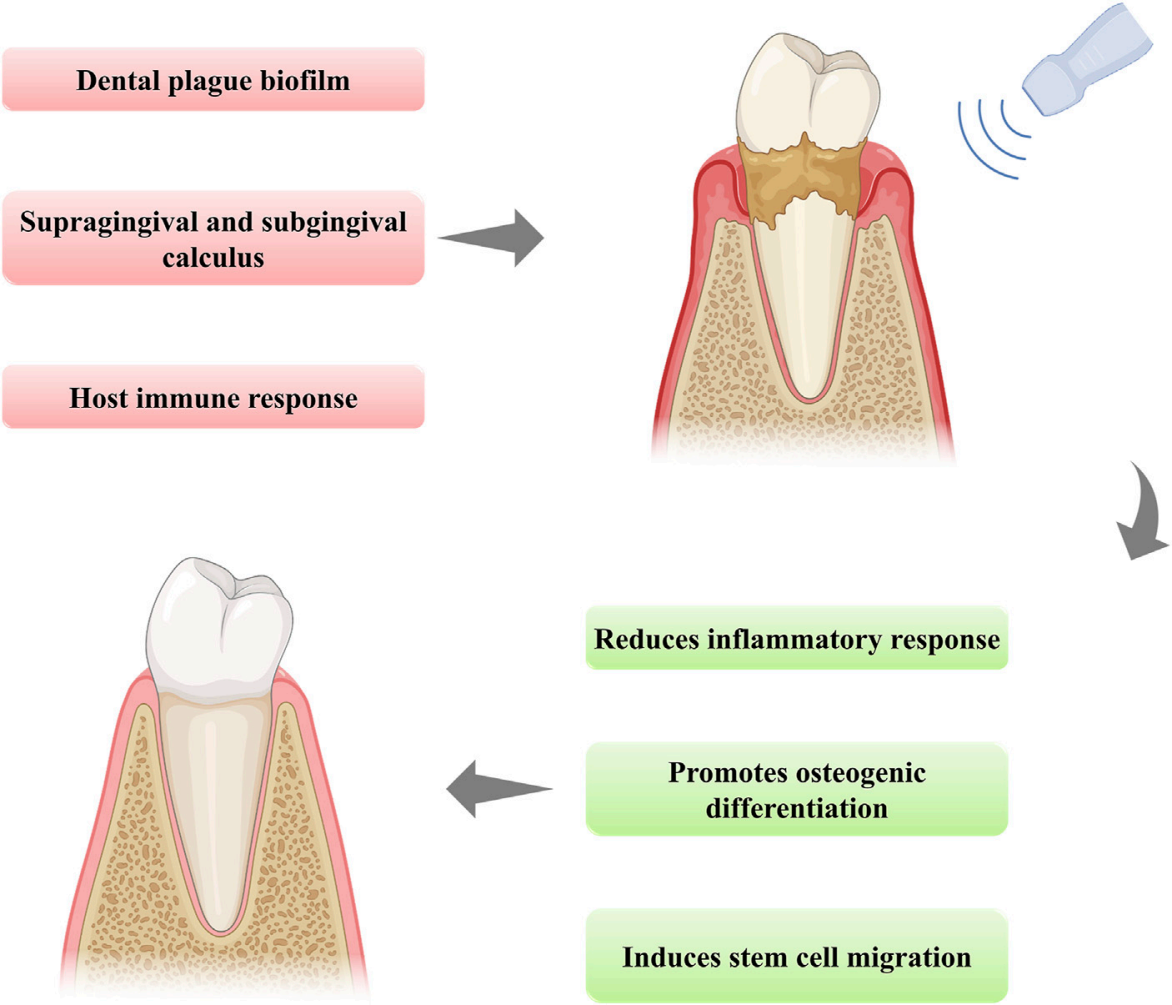
The schematic illustration of the function of LIPUS treatment in periodontitis.

Prolonged inflammation and periodontal bone destruction are the most severe consequences of periodontitis. LIPUS provides optimization for the existing advanced tissue regeneration technology in some experimental cases and also provides a new prospect for alveolar bone reconstruction in periodontitis (Figure 1). The mechanical stimulation provided by LIPUS can convert into biochemical signals and trigger downstream cascade reactions (Carina et al., 2018). Many studies have concentrated on the positive results and efficiency of LIPUS activation. However, before LIPUS therapy can be used in clinical periodontitis, the complete mechanism of its action in experimental periodontitis needs to be elucidated. In recent years, with the increasing attention to the treatment of LIPUS, more and more studies have focused on clarifying its therapeutic mechanism through its biological effects. The research of LIPUS in tissue regeneration as well as inflammation regulation has laid the foundation for studying its mechanism in periodontal tissue reconstruction. In light of the latest information available, the biological effects of LIPUS typically comprise the regulation of cell proliferation, migration, and differentiation *via* activating various molecular pathways. The precise mechanism by which mechanical stimuli are perceived and integrated into the periodontal regeneration process is not yet fully elucidated. Filling this vacancy can lead to enhanced treatment strategies for periodontitis and significantly shorten the duration of treatment. Therefore, this review aimed at summarizing and discussing the therapeutic mechanism of LIPUS and placing emphasis on how LIPUS manages to transfer mechanical stimulation into the signaling pathway to achieve inflammatory regulation and alveolar bone regeneration in experimental periodontitis. To push forward the progress of the mechanism study, we also point out the potential signaling pathways for further exploration.

## 2 Therapeutic mechanisms of LIPUS treatment

LIPUS has the potential for a wide range of applications, including bone healing, soft-tissue regeneration, inflammatory response inhibition, and neuromodulation, among others. The high-frequency pressure waves generated by LIPUS can produce mechanical stimulation in the targeted tissue and trigger biochemical events for tissue healing and regeneration. The involved therapeutic mechanisms for LIPUS-stimulated tissue healing have not been fully elucidated. Nevertheless, mechanical stress and/or fluid microfluidics are likely to be responsible for the biophysical effects of LIPUS (Tanaka et al., 2015). And when these events impact the cell plasma membrane, they trigger intracellular signal transduction and subsequent gene transcription.

### 2.1 LIPUS in bone regeneration therapy

As an active and dynamic tissue, bone is undergoing constant remodeling with proper biochemical and mechanical stimulations (Rowe et al., 2022). Appropriate mechanical stimulation of the bone is of crucial importance to preserve both the volume and structural stability of the bone. When the bone is subjected to mechanical loading, it promotes bone remodeling by enhancing the production and metabolism of osteoclasts and osteoblasts (Wang et al., 2022a). Various cellular activities, including those of cell proliferation, differentiation, gene expression, and protein synthesis, are activated and maintained by mechanical stimulation (Qin et al., 2020). Since the U.S. FDA approved the first LIPUS device for bone healing in 1994, extensive research has been carried out regarding fracture healing and presented desirable outcomes (Zura et al., 2015b). In 2018, Yusuf et al. reported a retrospective observational study that was conducted on clinical patients diagnosed with delayed or non-union, and 12 out of 18 patients successfully achieved full radiological union (Mirza et al., 2019). Subsequently, in 2019, Michael and his team performed a retrospective cohort study on non-unions of the distal upper limb treated with LIPUS (alone, or in conjunction with surgery). All patients underwent a low-intensity (30 mW/cm^2^) pulsed ultrasound treatment for 20 min/day over the fracture site for at least 3 months resulting in a final union rate of 62% overall (Elvey et al., 2020). These early studies provided the basis for in-depth research on LIPUS in the field of bone regeneration.

The use of LIPUS in bone fracture treatment appears to be successful at all phases of the fracture healing process, including regulating inflammation, accelerating vascularization, ossification, and eventually bone remodeling (Zhang et al., 2017a). The ability of LIPUS to influence different stages of bone healing indicates that LIPUS works through multiple mechanisms. As extensively shown *in vitro* studies, LIPUS increases the expression of genes related to bone formation, such as Runx-2, osteocalcin, TGF-β, collagen type I, and X, alkaline phosphatase (ALP), aggrecan, Insulin-like growth factor-1 (IGF-1) and bone sulfur protein (Pan et al., 2019b; He et al., 2021; Maung et al., 2021). Apart from that, LIPUS has also been described to promote protein synthesis and calcium absorption in different osteoblast cell lines (Tassinary et al., 2018). Mechanotransduction is the mechanism that has been put forward to explain the process where the mechanical signal is converted into a biological signal. This signal conversion is normally accomplished through mechanosensitive cells within the tissue. Notably, the main regulators of bone mechanosensation and mechanotransduction are osteocytes (Uda et al., 2017; Jiang et al., 2019; Shimizu et al., 2021). Osteocytes are the main moderators of bone homeostasis. They directly regulate local calcium concentration and indirectly control the osteoblasts and osteoclasts’ activities *via* the secretion of important regulatory factors. Shimizu et al. (2021) discovered that LIPUS stimulation alters gene expression patterns in osteoblasts. Osteoblasts promote the LIPUS-facilitated fracture healing through transcriptional regulation of the early growth response protein 1 and 2 (Egr1, and Egr2), forkhead box Q1 (FoxQ1), JunB, and nuclear factor of activated T-cells c1 (NFATc1). When mechanical stimulation induces fluid shear stress around osteocytes, integrins and kinase pathways act as mechanosensory to promote a series of cascade reactions (Geoghegan et al., 2019; McCarthy and Camci-Unal, 2021). Integrins activation results in the establishment of focal adhesions or focal contacts. Focal adhesions consist of several proteins including talin, paxillin, focal adhesion kinase (FAK), p130Cas, and vinculin (Stewart et al., 2020). According to research, LIPUS can phosphorylate FAK (Sang et al., 2021). As a consequence of the activated FAK, phosphor-inositol 3 kinase (PI3K) and protein kinase B (AKT) is subsequently phosphorylated, which triggers the integrin/phosphatidylinositol 3-OH kinase/Akt pathway (Zhang et al., 2016). In addition, the researchers revealed that the integrin/phosphatidylinositol 3-OH kinase/AKT pathway causes the formation of cyclooxygenase-2 (COX-2), which consequently raises prostaglandin-E2 (PGE2) level (Wang et al., 2018c), resulting in enhanced mineralization effect of osteoblasts (Harrison et al., 2016). Another major effect of LIPUS during bone healing progress is improved vascularization. Jacqueline et al. have proved that LIPUS upregulates the expression of vascular endothelial growth factor (VEGF) (Crossman et al., 2019).

### 2.2 LIPUS-induced anti-inflammatory effects

Inflammation plays a significant role in the tissue repair process, including timely activation and diminution. However, the prolonged inflammatory state will eventually cause destructive outcomes. In the last few decades, physical therapy has been used mostly in the field of rehabilitation. The outcomes of several studies in recent years have shown that physical therapy is also a promising anti-inflammatory approach (d’Agostino et al., 2015; Gardner et al., 2019). LIPUS functions in the regulation of inflammation by modulating the behavior of different cell types. Leukocytes play a critical role in the process of tissue healing. The inflammatory response is triggered immediately after injury, and leukocytes aggregate to clean up foreign material for inhibiting bacterial infection, which is beneficial to the subsequent tissue repair (Eming et al., 2017). As reported, this process can be regulated by mechanical stimulation like LIPUS by affecting the inflammatory infiltration stage (Rai et al., 2017). LIPUS has a bi-directional effect on leukocytes. LIPUS promotes the infiltration of white blood cells in the early stage of inflammation in repair, which helps clean the wounded area. While during the final phase of inflammatory repair, LIPUS reduces leukocyte infiltration, which will help prevent further tissue destruction (da Silva Junior et al., 2017). Feltham established the post-traumatic osteoarthritis (OA) model by creating an intra-articular fracture (IAF) in the right knee of rats and performed an experimental study. They reported a major decline in synovial leukocyte infiltration in the LIPUS-treated group in comparison to the control group (Feltham et al., 2021). In addition, LIPUS treatment decreased the infiltration of CD68^+^ macrophages in the synovium and the level of interleukin (IL)-1β in the joint fluid. Acoustic pressure waves have also been documented to affect the cell behavior of macrophages. For instance, in a rat model of hypothermic injury, da Silva Junior et al. (2017) showed that LIPUS lessened the number of inflammatory macrophages M1 after 1 day of treatment and increased the number of anti-inflammatory or reparative macrophages M2 after 2 days. We can conclude that LIPUS could alleviate persistent inflammatory responses by a decrease of M1 macrophages and enhancing tissue repair by increasing the M2 macrophages. These results well indicated the potential application of LIPUS in inflammatory diseases, while more research is required to explore the full mechanism.

Lipopolysaccharide (LPS) is associated with the pathogenesis of multiple diseases, such as osteoarthritis (Huang and Kraus, 2016), Alzheimer’s disease (Kim et al., 2021), coronary artery disease, and periodontitis (Liljestrand et al., 2017; Xu et al., 2020). And macrophages are among the target cells of LPS. In 2017, Zhang et al. (2017b) studied U937 macrophages and observed that LIPUS suppressed the expression of inflammatory factors induced by LPS, such as tumor necrosis factor-α (TNF-α), IL-1β, IL-6, and IL-8. They also discovered that this inhibition effect was activated by suppressing the toll-like receptor 4 (TLR 4)-nuclear factor κB (NF-κB) signaling pathway. Furthermore, LIPUS inhibited LPS-induced expression of nucleus pulposus (NP) inflammatory factors TNF-α and IL-1β also by obstructing the NF-κB signaling pathway. Fei et al. investigated the inflammatory response of an OA model developed in a C57BL/6 mouse with the anterior cruciate ligament transaction method. They discovered that as a consequence of activating focal adhesion kinase (FAK) signaling, LIPUS induced a decline in the expression of inflammatory cytokines TNF-α, IL-6, and IL-8 in the synovial fluid of OA mice (Sang et al., 2021). Zhang et al. (2020) also showed that LIPUS ameliorated synovial inflammation by inhibiting the secretion of mature IL-1β production in macrophages. And this process was achieved through the sequestosome-1 (SQSTM1) dependent autophagic degradation of pyruvate kinase isoenzyme type M2 (PKM). They also reported that LIPUS treatment attenuated the LPS-induced inflammatory response in RAW264.7 macrophages and reduced the LPS-induced increase in pro-inflammatory cytokines (TNF-α and IL-6) (Zheng et al., 2019). Their study was the first to demonstrate that LIPUS diminishes the active inflammatory response in acute viral myocarditis by activating caveolin-1 and suppressing the stimulation of mitogen-activated protein kinase (MAPK) signaling. LIPUS regulates inflammation by inhibiting inflammatory factors, however, LIPUS activates or suppresses different signaling pathways in different cell types.

### 2.3 Soft-tissue regeneration

It is reported that LIPUS treatment accelerates soft tissue regeneration, including tendon healing, ligament healing, intervertebral disc resorption, and cartilage recovery (Chen et al., 2019a; Li S. et al., 2021). This is especially because tissues such as tendons, ligaments, and cartilage have to bear a huge mechanical load in the body, and the application of LIPUS in the process of healing or tissue development can provide beneficial mechanical force. LIPUS has been reported to have beneficial effects on bone tendon healing by promoting fibroblast synthesis, collagen formation, and angiogenic, chondrogenic, and osteogenic activities (İnceoğlu et al., 2021). In a controlled study, an animal model of Achilles tendon injury has shown a more significantly increased in mature collagen fibers formation, improved biomechanical properties, and increased tissue regeneration rates under LIPUS treatment as opposed to the non-stimulation group (Lai et al., 2021). Human periodontal ligament cells (HPDLCs) play a fundamental role in periodontal regeneration. Low-intensity ultrasound had a similar effect on the activation of connective tissue cells, such as human osteoblast-like cell lines and HPDLCs (Jiang et al., 2019). There is also evidence from a recent study on a porcine model that the application of LIPUS on the oral mucosa expedites the repair of the masticatory mucosa (Chauvel-Picard et al., 2021). However, soft-tissue healing remains less studied than bone healing area. Like bone-healing research, LIPUS in soft tissue research still requires more randomized human clinical trials with controlled uniform parameters to obtain more systematic data to support alone and in combination with other stimulation methods.

### 2.4 Neuromodulation

The non-thermal neuro-modulatory effects of LIPUS were first reported by Tyler et al. (2008) demonstrating that LIPUS can remotely modulate neuronal circuits by activating action potentials and synaptic transmission. Many groups have established neuroprotective and reversible neuromodulation of LIPUS *in vitro* and *in vivo* since Tyler et al. (2008) published their results. Low-intensity ultrasound, like traditional pharmaceutical, electrical, magnetic, and optical treatments, can be used for functional neuromodulation. However, as distinguished from them, with a task-specific design ultrasound allows reversible, non-invasive neuromodulation with millimeter-level spatial resolution, without thermal effects (<0.01°) (Fini and Tyler, 2017). Lu et al. have successfully proved the positive role of LIPUS in preventing neuron degeneration in Parkinson’s disease (PD) by suppressing 1-Methyl- 4-phenylpyridinium (MPP+) evoked neurotoxicity and mitochondria malfunction (Zhao et al., 2017). They argued that pretreatment of PC12 cells with LIPUS would make contributions to protecting the cells from MPP + exposure (a major neurotoxic metabolite of PD) *via* modulating the antioxidative proteins to alleviate oxidative stress. They also pointed out several pathways as the underlying mechanism, for instance, the K2P channel and stretched-activated ion channel-mediated downstream pathways. The protective and regulatory effects of LIPUS on dopaminergic neurons were discussed in the most recent research (Chen et al., 2021). They developed PD models induced by 1-methyl-4-phenyl-1,2,3,6-tetrahydropyridine (MPTP) and MPP+ and explored the neuroprotective effect of LIPUS. This resulted that LIPUS exerts its neuroprotective effect by attenuating the central neurotoxicity of MPTP in mice, reducing the loss of tyrosine hydroxylase positive neurons in the substantia nigra pars compacta, and decreasing the apoptosis in the substantia nigra section. In addition, LIPUS was able to suppress MPP + -induced inhibition of dopaminergic neuronal activity and increase apoptosis and control the accretion of reactive oxygen species (ROS) and MPP + -induced decreases in membrane potential.

## 3 The mechanisms of LIPUS in treating experimental periodontitis

Based on the global burden of disease study in 2019, from 1990 to 2019, the global prevalence of periodontitis increased by 99.0% (Wu et al., 2022). The global burden of periodontitis has dramatically increased. Without adequate intervention, the chronically activated inflammatory state can lead to the progressive destruction of periodontal bone. Besides, periodontitis has been associated with diverse systemic diseases, including cardiovascular disease, diabetes, respiratory disease, and rheumatoid arthritis, according to several studies (Nazir, 2017). In recent decades, there are various treatment modalities available for periodontal regenerative therapy. Among them, regenerative surgery such as GTR has become the preferred solution to bone loss caused by periodontitis. However, infections due to foreign body reactions caused by bacteria or the implant material at the wound site continue to limit the clinical effectiveness of GTR technology (Lin et al., 2019). In addition, the success rate of the GTR procedure depends to a large extent on the operator’s surgical experience (Vaquette et al., 2018). Therefore, it is imperative to develop a method to accelerate periodontal regeneration. LIPUS has been increasingly studied in the field of tissue regeneration in recent years, and its mechanisms in bone regeneration and inflammation control have been considerably explored. Wang et al. (2022b) observed through an *in vivo* experimental study that LPIUS promotes new bone formation in the setting of periodontitis. In addition, they found that LIPUS is also effective in reducing inflammation and promoting angiogenesis, thus providing a favorable microenvironment for periodontal regeneration. Currently, LIPUS has shown advantages in both periodontal inflammation control and periodontal bone regeneration studies. However, there is still a lack of mechanism summarization and clarification in this area. In order to achieve more effective and precise clinical translation, it is necessary to fill this gap.

Previous studies have indicated that LIPUS can increase the proliferative activity of cells and enhance osteogenic differentiation (Baehni and Tonetti, 2010; Jiang et al., 2019). In terms of the periodontal field, studies have pointed out that LIPUS stimulation inhibits various inflammatory gene expressions and upregulates osteogenic differentiation-related genes (Tan et al., 2021). To elucidate the underlying mechanism of LIPUS in periodontitis, the periodontal cells were subjected to a specific ultrasound stimulation; however, loading parameters and time schedules varied among the studies (Table 1). Periodontal bone tissue originally possesses endogenous regenerative potential. There is evidence that a subpopulation subset of gingival fibroblasts (GFs), gingival mesenchymal stem cells (GMSCs), exhibit surface markers of mesenchymal stem cells (MSCs) and share an osteogenic differentiation potential similar to that of osteoprogenitor cells (Jin et al., 2015; Diar-Bakirly and El-Bialy, 2021). There has also been successful induction of osteogenic differentiation of GFs, which fully demonstrates the tissue regenerative potential of periodontal tissues (Mostafa et al., 2011; Liu et al., 2017). Another major cell type in periodontal connective tissue is periodontal ligament cells (PDLCs). PDLCs are a heterogeneous cell population inclusive of osteogenic progenitor cells and MSCs that can develop cementum, bone, and the periodontal ligament tissue itself (Yamamoto et al., 2018). Like bone marrow stromal cells, PDLCs can give rise to mesodermal and skeletal tissue regeneration. In the meantime, PDLCs also provide a beneficial microenvironment for bone deposition through the synthesis and release of growth factors, cytokines, colony-stimulating, and factors neurotransmitters (Chang et al., 2020). MSCs in the periodontal ligament, periodontal ligament stem cells (PDLSCs), also serve an important purpose in periodontal tissue regeneration (Zhai et al., 2019). Several *in vitro* studies have revealed the effect of varying intensities of LIPUS on the behavior of cementoblasts (Inubushi et al., 2008) and PDLCs, GFs, and osteoblasts (Inubushi et al., 2008). Nesrine et al. concluded that LIPUS can contribute to the osteogenic differentiation of GFs by upregulating the expression of osteogenic genes such as ALP and OCN (Mostafa et al., 2009). Studies have shown that LIPUS can improve the vitality, proliferation, migration, and multilineage differentiation of PDLSCs, and these effects of LIPUS may be regulated *via* various signaling pathways (Tan et al., 2021). In addition to this, LIPUS also exhibited a positive effect in enhancing the pluripotency of periodontal ligament cells. These characteristics have also motivated the study of LIPUS in periodontitis (El-Bialy et al., 2012). However, the related mechanisms still lack a systematic overview for a more effective clinical translation.

**TABLE 1:** Biological effects of LIPUS on periodontal ligament cells.

Cell types	Frequency (MHz)	Ultrasound intensity (SATA)	Treatment duration	Main outcomes	References
PDLFs	1.5	30 mW/cm^2^	20 min/day	Suppresses inflammatory effects of LPS-PG, IL-1β, and TNF-α	Augustine et al. (2021)
				Promote BMP9-induced osteogenesis through ROCK1	
PDLCs	1.5	90 mW/cm^2^	30 min/day	Inhibits IL-6 and IL-8 gene expression	Leewananthawet et al. (2019)
				Increases expression of osteogenic markers (Runx2, OPN, OSX, and OCN)	
				Inhibits NF-κB signaling pathway	
PDLCs	1.5	90 mW/cm^2^	20 min/day	Increases mRNA expression of BSP, COL-3, OPN, and calcium deposition	Liu et al. (2020)
PDLSCs	1.0	250 and 750 mW/cm^2^	5, 20 min/day	Increases proliferation	Gao et al. (2017)
				Immediate activation of JNK, MAPK	
				Significant increase in phosphorylated p38 MAPK	
PDLSCs	1.5	30, 60 and 90 mW/cm^2^	30 min/day	Increases cell migration	Kechagia et al. (2019)
				Increases the mRNA and protein levels of TWIST1 and SDF-1	
PDLCs	1.5	90 mW/cm^2^	20 min/day	Increases osteogenic differentiation (ALP, OCN) and mineralization	Xu et al. (2020)
				Increases expression of Runx2 and integrin β1	
PDLCs	1.5	90 mW/cm^2^	30 min/day	Increases expression of osteogenic markers (ALP, Runx2) and matrix mineralization	Chen et al. (2019c)
				Inhibits miR-182 expression and promotes FOXO1 accumulation	
PDLCs	1.5	90 mW/cm^2^	2 h	Increases mRNA and protein expression of autophagic genes Beclin-1 and LC3	Ying et al. (2020)
				Decreases IL-6 expression	
PDLSCs	1.5	90 mW/cm^2^	30 min/day	Increases proliferation, matrix mineralization and osteogenic differentiation (ALP, Runx2)	Li et al. (2020a)
				Decreases IL-6 and IL-8 expression	
				UPR pathway involved	
PDLSCs	1.5	90 mW/cm^2^	30 min/day	Increases cell proliferation, matrix mineralization and expression of Runx2, OPN, OCN, COL-1, ALP, integrinβ-1	Li S. et al. (2021)

SATA, spatial average temporal average; PDLFs, periodontal ligament fibroblasts; PDLCs, periodontal ligament cells; PDLSCs, periodontal ligament, periodontal ligament stem cells; LPS-PG, porphyromonas gingivalis lipopolysaccharide; IL, interleukin; TNF-α, tumour necrosis factor alpha; BMP, bone morphogenetic protein; ROCK1, rho-associated kinase 1; Runx2, runt-related transcription factor 2; OPN, osteopontin; OSX, osterix; OCN, osteocalcin; NF-κB, nuclear factor-κB; BSP, bone sialoprotein; COL, collagen; JNK, c-Jun NH2-terminal kinase; MAPK, mitogen-activated protein kinases; TWIST1, twist family bHLH, transcription factor 1; SDF-1, stromal cell-derived factor 1; ALP, alkaline phosphatase; FOXO, forkhead box O; LC3, light chain 3; UPR, unfolded protein response.

### 3.1 LIPUS induces stem cells migration

The mechanical stimuli in the process of bone remodeling realize the balance of regeneration and absorption of bone tissue through mechanotransduction (Ascolani et al., 2021). The role of mechanotransduction in periodontal regeneration was reflected in regulating cell migration (Wang et al., 2018b), proliferation (Huelter-Hassler et al., 2017), differentiation (He et al., 2019; Papadopoulou et al., 2020), and suppressing inflammatory factors *via* mechanosensory elements within cells. Restorative cell migration is a critical step in periodontal tissue repair. Endogenous MSCs have been shown to enhance tissue healing by homing to the site of injury (Lin et al., 2017). Previous studies have shown that BMSCs are motivated by a specific chemotactic factor released at the fracture site, thus migrating to the damaged area, and differentiating into osteoblasts to repair the fracture (Zhang et al., 2018). In addition, the mobilization of MSCs is involved in the homeostasis of periodontal tissues. Thus, the homing of stem cells from the surrounding healthy periodontal tissue into remodeling sites would presumably play an important role during *in situ* periodontal bone regeneration.

**FIGURE 2:**
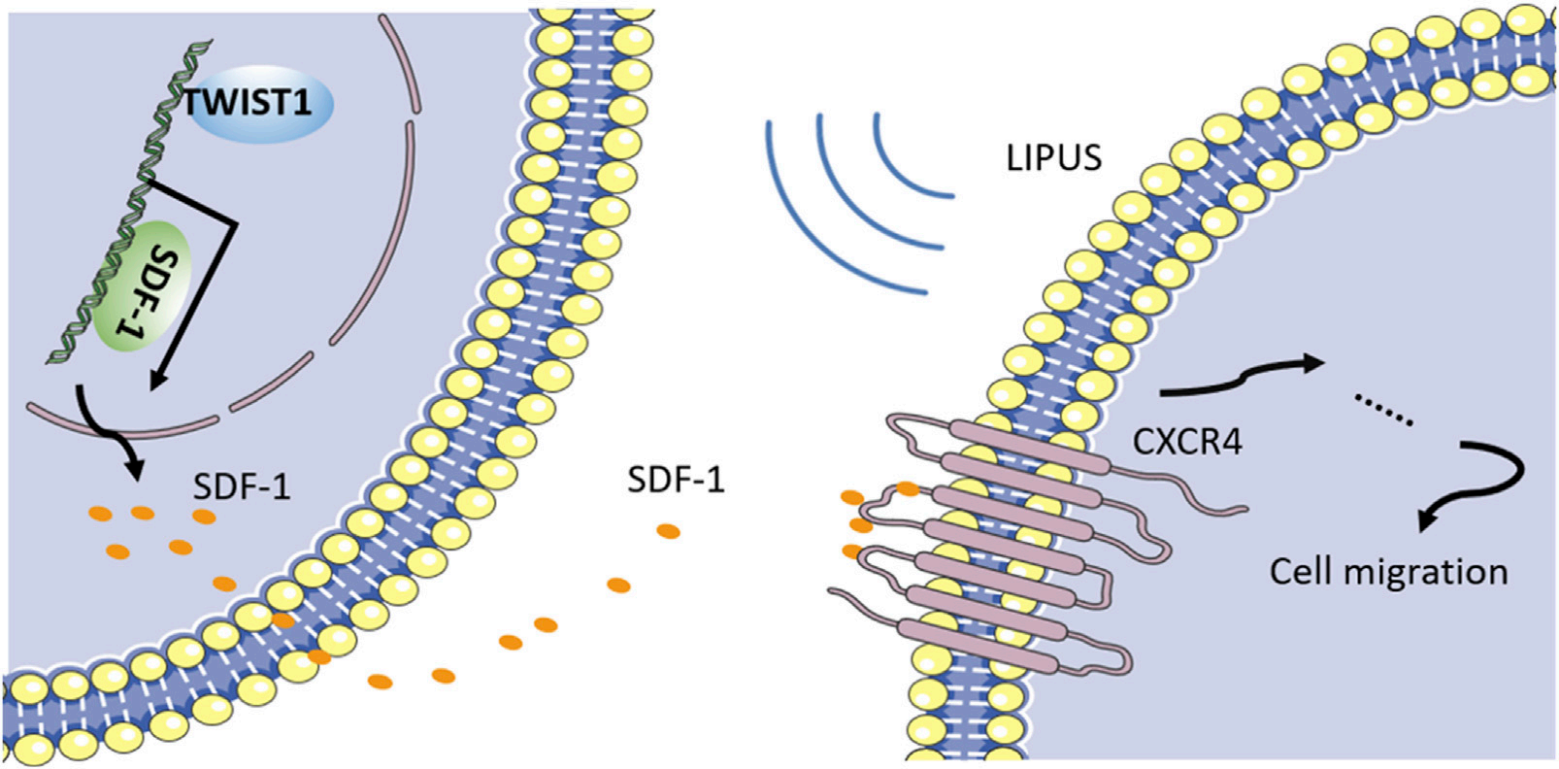
LIPUS promotes PDLSCs migration through SDF-1/CXCR4 signaling pathway.

Research has gathered evidence that homing of PDLSCs is a potential mechanism of LIPUS-mediated periodontal tissue regeneration. PDLSCs are mechanosensitive cells and have an essential effect on tissue homeostasis and repair (Men et al., 2020). Previous studies showed that LIPUS possesses the ability to accelerate fracture healing by activating the migration of osteogenic progenitors to the damaged sites. Also, stromal cell-derived factor 1 (SDF-1), a type of chemokine that is vital for stem cell homing, is upregulated in the fracture site under LIPUS stimulation. To further investigate, WANG et al. conducted experiments on PDLSCs and revealed that LIPUS treatment facilitates the gene and protein expression of SDF-1 in PDLSCs (Wang et al., 2018b). Blocking SDF-1 or its receptor, C-X-C motif chemokine receptor 4 (CXCR4), significantly inhibits the secretion of SDF-1 and LIPUS-induced cell migration. These results indicate that the SDF-1/CXCR4 pathway is an important molecular mechanism underlying the LIPUS-induced stem cell migration. However, how mechanical signals are sensed and thus cause the activation of this signaling pathway? Recently, twist family bHLH transcription factor 1 (TWIST1) is regarded as having a fundamental role in the remodeling of the alveolar bone-periodontal ligament interface. Besides TWIST1 was reported to increase SDF-1 expression in a dose-dependent modality. WANG et al. further verified the role of TWIST1 in PDLSCs. They discovered that the knockdown of TWIST1 not only suppressed the LIPUS-induced SDF-1 expression but also blocked the LIPUS-induced stem cell migration. Therefore, we have concluded that TWIST1 acts as a mechanical stress sensor during mechanotransduction. When periodontal defects are treated with LIPUS stimulation, TWIST1 in PDLSCs will be activated and induce SDF-1 secretion. Eventually, LIPUS promotes PDLSC homing *via* the SDF-1/CXCR4 pathway to achieve periodontal bone regeneration (Figure 2). Naturally, cell migration is dependent on the involvement of adhesion proteins such as integrins and FAK activation. There is also strong evidence that LIPUS can regulate the migration of BMSCs by activating the FAK-ERK signaling pathway (Chen et al., 2019d). However, studies of this pathway in LIPUS-promoted migration of PDLCs are still absent.

However, the migration of PDLCs was not completely abolished by the blockade of TWIST1, suggesting that the existence of compensatory mechanisms may be possible. MSC migration is enhanced by the expression of additional chemokine receptors such as CCR1, CCR4, and CCR7, according to a previous study. As a result, the processes by which MSCs are mobilized to periodontal tissues in response to LIPUS are yet to be fully explored.

### 3.2 LIPUS promotes stem cells osteogenic differentiation

Human periodontal ligament cells (HPDLCs) possess various differentiation abilities and can facilitate the regeneration of periodontal tissues by differentiating into osteoblasts and cementoblasts (Zhu and Liang, 2015). The osteogenic differentiation capacity of HPDLSCs plays an essential role in the process of periodontal bone tissue reconstruction. The application of LIPUS can enhance the osteogenic differentiation of HPDLSCs by regulating related signaling pathways (Li H. et al., 2021). MAPK signaling pathway is an important mechanism underlying the osteogenic differentiation of HPDLSCs (Wang et al., 2018a). Mammalian MAP kinases are comprised of the extracellular signal-regulated kinase (ERK) family, the p38 kinase family, and the c-Jun N-terminal kinase family (JNK, also known as stress-activated protein kinase or SAPK) (Roskoski, 2012). Ren et al. (2013) confirmed that the p38 MAPK signaling pathway participates in the LIPUS-induced osteogenic differentiation of HPDLCs through the significant increase in LIPUS-induced ALP secretion, osteocalcin production, and calcium deposition (Huang et al., 2018). In a similar study, Gao et al. (2016) used specific MAPK inhibitors that further confirmed the role of specific MAPK pathways in the PDLSC proliferative response to ultrasound. They found that JNK and p38 may be both involved in the stimulation of PDLSC proliferation. Later in 2017, they also confirmed that phosphorylated JNK and p38 MAPK were increased mainly in PDLSCs over 24 h after LIPUS stimulation (Gao et al., 2017).

It is understood that LIPUS generates mechanical stresses, which can affect specific cellular mechanical transduction components, such as integrins, focal adhesion complexes, membrane receptors, ion channels, and cytoskeleton components. Integrins, which connect the cytoskeleton to the extracellular matrix and mediate a variety of signaling cascades, are also involved in the transduction of mechanical stimuli to biochemical signals (Kechagia et al., 2019). When LIPUS signals are delivered to integrins, the binding of varied adhesion junction proteins is triggered. One of the crucial focal adhesion proteins in the conversion of LIPUS signaling from mechanical to biochemical signals is focal adhesion kinase (FAK), which regulates integrin-mediated signaling triggered by LIPUS (Zhang et al., 2017a). Integrin β1 is implicated in the remodeling of the periodontium in response to mechanical stimulation as a mechanoreceptor on the cell membrane (Molina et al., 2001). Hu et al. (2014) proved that integrin β1-dependent signaling transduction was included in the LIPUS-induced osteogenic differentiation of HPDLCs. When the mechanical signals from LIPUS reach the HPDLCs, the integrin β1-dependent signaling pathway will be activated and presumably activates the MAPK pathway. Finally, LIPUS facilitates Runx2 expression, ALP secretion, osteocalcin production, and calcium deposition in HPDLCs.

HPDLCs can initiate osteogenic differentiation *via* stimulation of cytokines and growth factors such as platelet-derived growth factor (Yang et al., 2022), epidermal growth factor, transforming growth factor (Li and Zhang, 2015), and bone morphogenetic protein (BMP) (Khanna-Jain et al., 2010). Of those factors, BMP has been identified as a key mediator in osteoblast differentiation regulation. It was shown that LIPUS plays a significant role in the BMP-induced osteogenic differentiation of PDLCs. Yang et al. (2014) discovered in their early study that BMP-2 and BMP-6 expression in HPDLCs increased after daily LIPUS treatment. They also explored that this process is achieved through the BMP-Smad signaling pathway. As an early response to mechanical signals generated by LIPUS, Smad transcription factors in PDLCs are phosphorylated on serine residues by the BMP receptor complex. Smads are categorized into 3 classes: receptor-regulated Smads, common Smads, and inhibitory Smads, and each of them has a particular function (Miyazawa and Miyazono, 2017). Once the receptor-regulated Smads (Smad1/5/8) are phosphorylated, receptor-regulated Smads dissociate from the receptor, connect to common Smad (Smad4), and enter the nucleus. In the nucleus, heteromeric Smad complexes function as effectors of BMP signaling by mediating transcription-related genes to upregulate multiple osteogenic differentiation makers.

**FIGURE 3:**
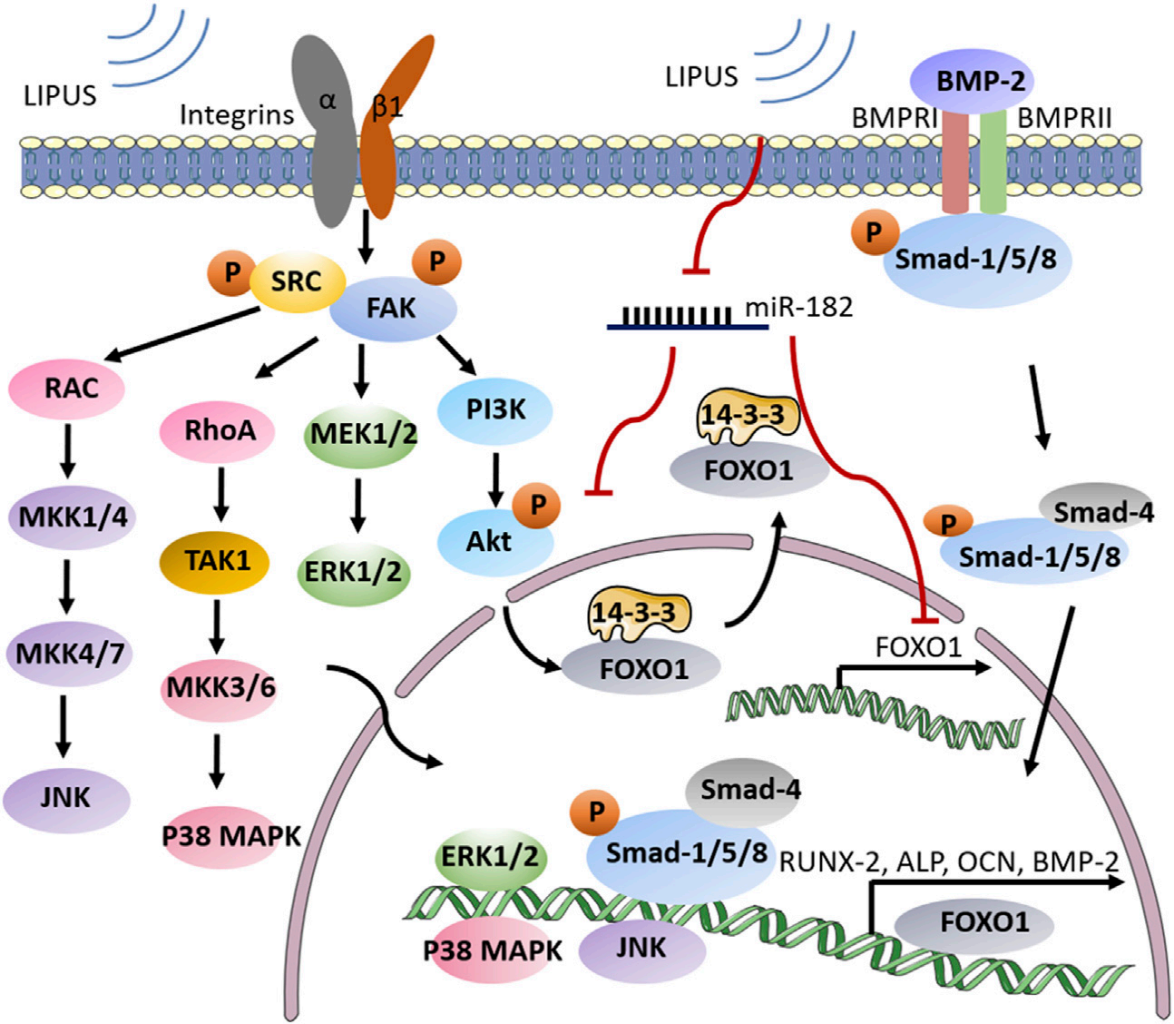
Schematic illustration of LIPUS enhancing osteogenic differentiation in periodontal tissue. LIPUS promotes FAK phosphorylation through integrin β1 and subsequently activates MAPK signaling, enabling the transcription of osteogenic-related signaling factors. Meanwhile, LIPUS can reverse the inhibitory effect of miR-182 on FOXO1 by inhibiting it, and prevent the overexpression of FOXO1 through PI3K/Akt pathway. The BMP receptor complex is also activated by LIPUS stimulation and promotes the expression of RUNX-2, ALP, OCN and other osteogenic genes through the BMP-Smad signaling pathway.

In bone tissue, FOXO (Forkhead box O), a large family of forkhead transcription factors plays a crucial role in osteogenesis (Chen et al., 2019b). Some scholars have also investigated the mechanism of FOXO1 involvement in LIPUS-promoted osteogenic differentiation of HPDLCs. By knocking down the FOXO1, Chen et al. (2019c) discovered a major decrease in the level of osteogenic differentiation markers (like ALP, and Runx2) in HPDLCs. A collection of FOXO1-sensitive miRNAs, including miR-182, miR-183, and miR-705, have been revealed to be important regulators of osteogenic differentiation (Pitto et al., 2008; Wang et al., 2016). On this basis, Chen et al. (2019c) also found that LIPUS stimulation could inhibit miR-182 and thus attenuate its inhibitory effect on FOXO-1 accumulation. PI3K/Akt signaling pathway is another basic and traditional level of FOXO1 activity regulation, in addition to miRNA-mediated post-transcriptional regulation (Xing et al., 2018). LIPUS was discovered to induce Akt phosphorylation, which prevented active FOXO1 excessive accumulation by driving nucleus FOXO1 translocation to the cytoplasm and helping to maintain the balance of FOXO1 activity. Phosphorylated-Akt produced two 14-3-3 protein binding sites on FOXO1, and the FOXO1-14-3-3 binding complex was transported from the nucleus to the cytoplasm, where the bound 14-3-3 protein inhibited FOXO1 from returning to the nucleus. In osteoblasts, FOXO1 was revealed to be phosphorylated at Thr-24, Ser-256, and Ser-319 *via* the PI3K/Akt pathway (Fukunaga et al., 2005), while the phosphorylation sites in HPDLCs subject to LIPUS were unknown. In addition, miR-182 was discovered to retain FOXO1 in the nucleus in human hepatic cells by reducing Akt phosphorylation, indicating the intricacy and potential balance capacity of miR-182 on FOXO1 activity. Taken together, Chen et al. have concluded that miR-182 converts LIPUS stimulation into the biochemical signal FOXO1 and contributes to enhanced osteogenic differentiation in HPDLCs. Besides, LIPUS increases cytoplasm translocation of FOXO1 *via* PI3K/Akt pathway. However, the potential role of miR-182 in LIPUS-mediated Akt phosphorylation within HPDLCs is subject to further study. The mechanism by which LIPUS regulates the osteogenic differentiation of PDLCs is elucidated in Figure 3.

### 3.3 LIPUS modulates inflammatory response in periodontitis

**FIGURE 4:**
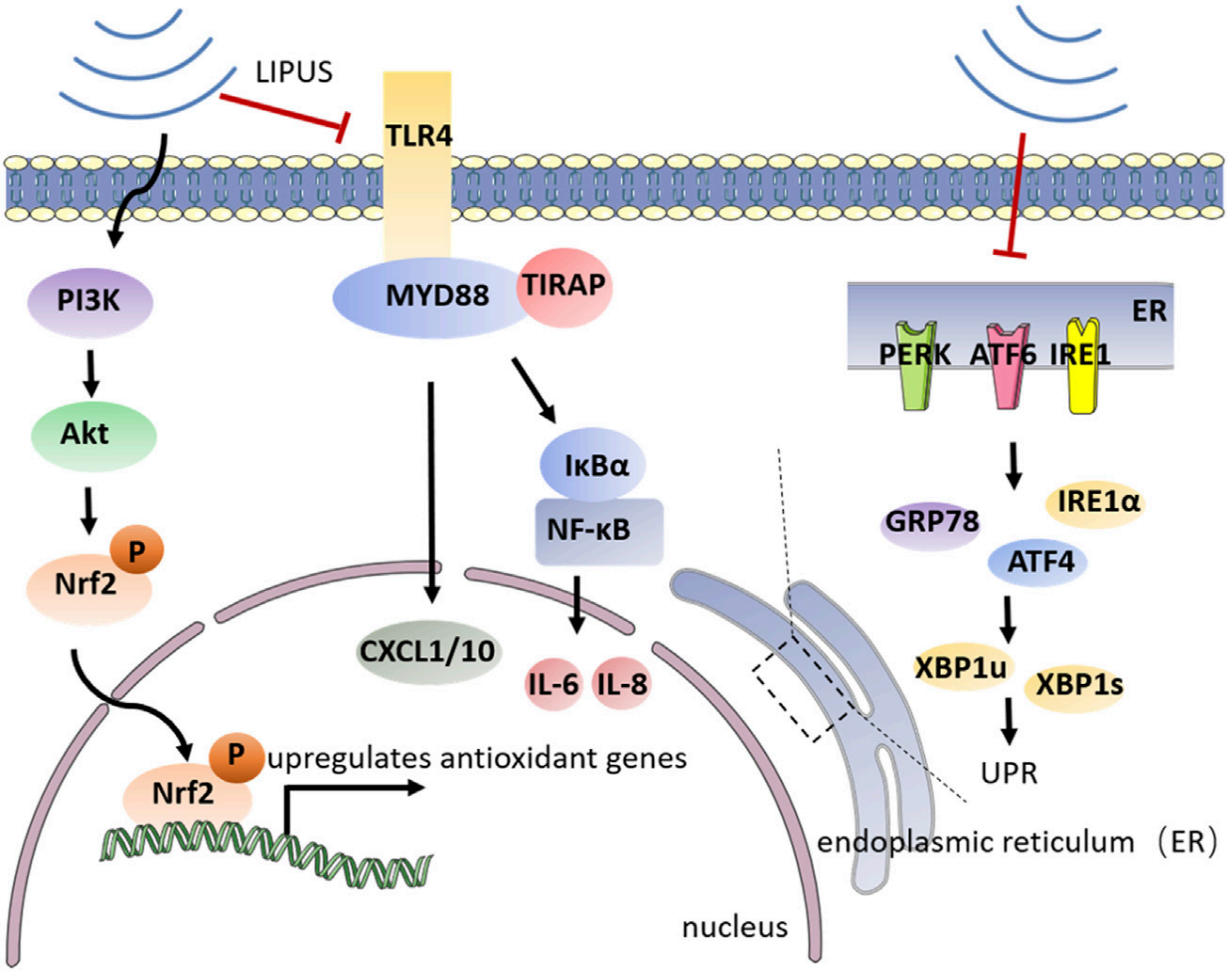
Schematic illustration of the mechanism by which LIPUS regulates periodontal inflammation. LIPUS can release UPR and reduce the secretion of chemokines such as IL-6, IL-8, and CXCL1/10 by inhibiting TLR4-mediated NF-κB signaling pathway and specific transmembrane signaling transducers on the endoplasmic reticulum surface. LIPUS can also activate the PIS/Atk signaling pathway to further upregulate antioxidant genes and alleviate oxidative stress.

Dental plaque biofilm is the initiating factor of periodontitis, host inflammatory and immune responses to microorganisms inside the biofilm can leave the subgingival environment in a state of ischemia and hypoxia. The anaerobic environment causes *Porphyromonas gingivalis (P. gingivalis)*, the key pathogen underlying progressive periodontitis and severe periodontitis, to become the dominant bacterium in the biofilm. As an important virulence factor in (*P. gingivalis*), *lipopolysaccharide* (LPS) is able to stimulate signaling factors such as Toll-like receptor-4 (TLR4) leading to unwanted host inflammatory responses. An inflammatory state will greatly weaken the self-renewal and multiple differentiation ability of periodontal tissue. NF-κB pathway has an important part in the TLR-mediated secretion of pro-inflammatory signals (cytokines, chemokines, and adhesion molecules) (Xu et al., 2020). As a major pathogenic factor in exacerbating periodontitis, LPS can increase periodontal inflammation by activating the intracellular NF-κB signaling pathway through TLR4. Activation of the NF-κB signaling pathway, on the other hand, impairs the osteogenic differentiation of PDLSCs. The microenvironment of periodontitis has a great impact on the osteogenic differentiation ability of cells. The persistent inflammatory condition arrests the differentiation of osteoblasts and increases the numbers and activity of osteoclasts. Oxidative stress is considered one of the pathophysiological mechanisms driving periodontitis, according to numerous research (Sczepanik et al., 2020). Oxidative stress hinders the osteogenic differentiation of PDLCs and reduces their regenerative potential (Kuang et al., 2020). Furthermore, the inflammatory state imposes endoplasmic reticulum stress (ERS), a pathological state that invokes an intracellular unfolded protein response (UPR), on PDLSCs leading to decreased osteogenic differentiation (Qiao et al., 2021; Tan et al., 2022). ERS is actuated by the accretion of unfolded and misfolded proteins in the endoplasmic reticulum (ER) (Wang et al., 2016). During ERS, cells react to changes in protein folding by activating a response called UPR. Up until now, UPR is initiated through three ER transmembrane transducers, including protein kinase R-like ER kinase (PERK), activating transcription factor 6 (ATF6) and inositol acquisition enzyme 1 (IRE1) (Ghemrawi and Khair, 2020; Read and Schröder, 2021). However, LIPUS provides more targeted inhibition of these pro-inflammatory mechanisms. LIPUS suppresses *lipopolysaccharide (LPS)*-induced inflammatory chemokines, diminishing oxidative stress and UPR, protecting periodontal osteogenic differentiation potential while regulating inflammation-related signaling pathways (Figure 4).

NF-κB signaling pathway is closely involved in periodontal inflammation. TLR4/MyD88/NF-κB signaling played an important role in the LPS-induced inflammatory response of periodontal tissue. Nakao et al. (2014) showed that LIPUS interferes with the TLR4-MyD88/TIRAP signaling pathway in osteoblasts, thereby inhibiting the gene expression of the LPS-induced chemokines CXCL1 and CXCL10. And Liu et al. suggested that LIPUS reduces the inflammatory factors of U937 and potentiated the viability and osteogenic differentiation of PDLC *in vitro* (Zhang et al., 2017b). A recent study conducted by Liu et al. (2020) demonstrated with persuasive evidence that LIPUS inhibits the NF-κB signaling pathway by preventing the phosphorylation of IκBα and translocation of p65 into the nucleus. Therefore, LIPUS inhibits the secretion of IL-6 and IL-8 and modifies the osteogenic differentiation potential of hPDLCs in an inflammatory environment. However, the results of this experiment do not clarify whether LIPUS has a time-dependent effect on anti-inflammation, which needs further improvement. However, the results of this experiment did not elucidate whether LIPUS has an anti-inflammatory time-dependent property, which needs further improvement. Also, whether the duration of the LIPUS application plays a role in the alteration of signaling pathways needs further experimental validation. In addition, (Kusuyama et al. (2019) have also discovered that LIPUS treatment significantly suppressed LPS-induced mRNA expression of IL family cytokines, RANKL, and chemokines in periodontal ligament fibroblasts (PDLFs). And they explored that ROCK1, a molecule that mainly participated in cytoskeletal rearrangement after Rho activation, is involved in the suppression of inflammatory response by LIPUS.

As an effective treatment, LIPUS can effectively regulate inflammation, thus providing an improved microenvironment for the osteogenic differentiation of cells. It was found that LIPUS stimulation was effective in reducing oxidative stress in periodontitis and alleviating the inhibition of osteogenic differentiation and alveolar bone destruction caused by oxidative stress. (Ying et al. (2020) demonstrated that LIPUS acts to protect alveolar bone from oxidative stress by upregulating and activating nuclear factor erythroid 2-related factor 2 (Nrf2). Nrf2, as a redox-sensitive transcription factor, derives upregulation of a series of antioxidant genes that protect tissue against oxidative stress (Leewananthawet et al., 2019; Li et al., 2019). PI3K/Akt is a widely recognized upstream regulator of Nrf2, and activation of PI3K/AKT directly reduces the degradation of Nrf2 by facilitating its phosphorylation and accelerating its translocation to the nucleus (Li et al., 2018). By utilizing a pharmacological inhibitor of the PI3K/Akt pathway, Ying et al. (2020) also exhibited that the PI3K/AKT pathway promotes LIPUS-mediated upregulation of nuclear Nrf2. Therefore, it can be understood that LIPUS can effectively protect against tissue damage due to oxidative stress in experimental periodontal inflammation.

PDLSCs are a set of MSCs with multidirectional differentiation potential. They can serve an important purpose in the reconstruction of periodontal bone tissue in a healthy periodontal state. However, the intracellular ERS state caused by the inflammatory microenvironment severely affects the osteogenic differentiation potential of stem cells, thus leading to increased bone loss after periodontitis. Li et al. (2020a) demonstrated that through the inhibition of ERS, LIPUS reduces inflammation and facilitates osteogenesis in the LPS-induced inflammatory environment. And the UPR-related genes IRE1α, GRP78, and ATF4 were also downregulated after LIPUS treatment. LIPUS treatment also suppressed the expression of XBP1u and XBP1s, the target genes of IRE1α in the UPR pathway. Meanwhile, LIPUS increased the expressions of RUNX2 and ALP, indicating that LIPUS improves the osteogenic differentiation potential of PDLSCs *via* inhibiting UPR. While the specific pathway included in the use of LIPUS and the regulation of the UPR still need so further investigation.

Autophagy is a catabolic process in which cells decompose their unneeded proteins, macromolecular complexes, and organelles, passing them into lysosomes so and enabling them to survive (Saha et al., 2018). Clinical studies show that autophagy is a requirement in the inflammatory microenvironment to protect PDLSCs from apoptosis (An et al., 2016). Li et al. (2020b) have demonstrated that LIPUS stimulation can induce mRNA and protein expression of the autophagy pathway related-proteins, such as LC3 and Beclin-1, in LPS-pretreated HPDLCs. LIPUS pretreatment significantly reduces IL-6 release in lipopolysaccharide-stimulated HPDLCs. However, the autophagy inhibitor, 3-Methyladenine, causes an increase in the level of IL-6, which indicates the involvement of autophagy in the LIPUS anti-inflammatory mechanism in HPDLCs.

## 4 Summary and prospects

Periodontitis is a widespread oral disease that causes alveolar bone destruction. If properly contained, early-state periodontitis has a good prognosis. However, severe bone loss, a complication of more advanced periodontitis, is still a puzzle for clinicians. LIPUS provides a new inspiration for tissue regeneration. In contrast to high-intensity ultrasound applied for tissue heating, LIPUS provides mainly non-thermal effects, including microbubbles and microjets caused by cavitation, acoustic flow, mechanical stimulation, etc. and in this article, we thoroughly discussed the underlying mechanism of LIPUS-mediated periodontal bone regeneration. As a mechanical signal, LIPUS activates different signaling pathways *via* mechanotransduction and subsequently regulates cell behavior. LIPUS can activate the homing of endogenous MSCs as well as osteogenic progenitor cells to the defected area by activating the TWIST/SDF-1 signaling pathway. When LIPUS reaches the damaged tissue, intracellular TWIST1 is activated to promote the expression of SDF-1, which in turn serves to promote the expression of genes related to cell migration through its corresponding receptors. However, studies on how periodontal ligament cells perceive mechanical signals from LIPUS and translate them into the expression of signaling molecules that promote migration remain imperfect. Besides, LIPUS regulates the osteogenic differentiation of PDLCs through the involvement of signaling pathways such as MAPK, BMP-Smad, and cytokines such as FOXO. In periodontal tissue, integrin β1 functions as a sensory transducer of mechanical stimulation from LIPUS, promoting FAK phosphorylation and thereby activating a series of downstream cascade responses. MAPK signaling pathway activated by phosphorylation of FAK increases the expression of ERK1/2, p38 MAPK, and JNK and thus contributes to the transcription of osteogenic differentiation genes. LIPUS increases the expression of Runx2, ALP, and the mineralized bone nodule formation by promoting the transcriptional function of FOXO1. By inhibiting the FOXO1-sensitive miRNA miR-182, LIPUS ensured the normal transcription of FOXO1 in the nucleus. In this process, the translocation of FOXO1 from the nucleus to the cytoplasm can be achieved through the PI3K/Akt signaling pathway thus avoiding the excessive accumulation of FOXO1 in the nucleus. In addition, LIPUS upregulates osteogenic differentiation gene markers in periodontal cells by enabling the nuclear transport of the Smad complexes through the BMP-Smad signaling pathway. In the meantime, LIPUS generates mechanical signals that inhibit the release of inflammatory factors, oxidative stress, and UPR activated by periodontitis, and modulates periodontal inflammation by regulating cellular autophagy. When exposed to LIPUS, the signaling pathway promoting antioxidant gene transcription was activated, while the TLR-mediated NF-κB and UPR-related signaling pathways associated with pro-inflammation were inhibited. LIPUS phosphorylates transcription factor Nrf2 through PI3K/Akt signaling pathway and promotes antioxidant genes to reduce oxidative stress and periodontitis-induced tissue damage. LIPUS also suppressed the TLR-mediated NF-κB signaling pathway, while interfering with the TLR4-MyD88-TIRAP complex to reduce the release of LPS-induced chemokines. In addition, LIPUS could inhibit UPR-induced inflammatory response by suppressing the ER transmembrane transducers and their downstream genes.

Nevertheless, the mechanism of LIPUS-induced periodontal bone tissue regeneration remains incomplete. Although the current study can describe the general mechanism of the effect of LIPUS on periodontal bone regeneration, however, many details need to be refined. The current research had not proposed the optimal LIPUS stimulation parameters corresponding to various cells. Different intensities and stimulation durations may reveal different signaling pathways of mechanotransduction, so we believe that the research on LIPUS needs to be further improved on this aspect to obtain more rigorous results. LIPUS gradually possesses a more significant place in the field of regeneration medicine. And a thorough knowledge of the molecular mechanism behind LIPUS-related treatments will pave the way for better clinical application.
